# A molecular and morphological study of *Ascaris suum* in a human-pig contact scenario in northeastern Brazil

**DOI:** 10.1590/S1984-29612023057

**Published:** 2023-10-13

**Authors:** Polyanna Araújo Alves Bacelar, Jéssica Pereira dos Santos, Deiviane Aparecida Calegar, Denilson de Araújo e Silva, Daniella Nobre Leal, Brenda Bulsara Costa Evangelista, Elis Regina Chaves dos Reis, Jacenir Reis dos Santos Mallet, Filipe Anibal Carvalho-Costa, Lauren Hubert Jaeger, Kerla Joeline Lima Monteiro

**Affiliations:** 1 Laboratório de Epidemiologia e Sistemática Molecular, Instituto Oswaldo Cruz, Fundação Oswaldo Cruz - FIOCRUZ, Rio de Janeiro, RJ, Brasil; 2 Laboratório de Parasitologia Molecular, Escritório Regional Fiocruz Piauí, Fundação Oswaldo Cruz - FIOCRUZ, Teresina, PI, Brasil; 3 Secretaria Municipal de Saúde, Nossa Senhora de Nazaré, PI, Brasil; 4 Laboratório de Vigilância Entomológica em Díptera e Hemíptera, Instituto Oswaldo Cruz, Fundação Oswaldo Cruz - FIOCRUZ, Rio de Janeiro, RJ, Brasil; 5 Departamento de Ciências Farmacêuticas, Faculdade de Farmácia, Universidade Federal de Juiz de Fora - UFJF, Juiz de Fora, MG, Brasil

**Keywords:** Ascaris suum, Sus scrofa domesticus, cytochome oxidase 1, phylogenetic analyses, Ascaris suum, Sus scrofa domesticus, citocromo oxidase 1, análise filogenética

## Abstract

The aim of the present study was to assess morphologic and genetic data on ascariasis in swine (*Sus scrofa domesticus*) and humans in low-resource rural and periurban communities in the state of Piauí, Brazil. Our cross-sectional survey included 100 fecal samples obtained from swine and 682 samples from humans. Fifteen pigs were necropsied. Human and porcine fecal samples were examined to identify *Ascaris* eggs. Parasites obtained in the swine necropsies were studied using scanning electron microscopy (SEM), and the mitochondrial gene encoding the cytochrome oxidase 1 (*cox*1) enzyme was partially amplified and sequenced for molecular taxonomy and phylogenetic analyses. The overall prevalence of *Ascaris* eggs in the swine fecal samples was 16/100 (16%). No *Ascaris* eggs were identified in the human fecal samples. SEM of six worms recovered from pigs demonstrated morphological characteristics of *A. suum*. *Cox*1 sequences were compatible with *A. suum* reference sequences. Original and reference (GenBank) nucleotide sequences were organized into clusters that did not segregate the parasites by host species or and region. The largest haplogroups were dominated by haplotypes H01, H02 and H31. In the communities studied, there was no epidemiological evidence of the zoonotic transmission of ascariasis at the human-swine interface.

## Resumo

O presente estudo teve como objetivo acessar dados morfológicos e genéticos sobre a ascaridíase em suínos (*Sus scrofa domesticus*) e humanos, em comunidades rurais e periurbanas no estado do Piauí. O estudo transversal incluiu 100 amostras fecais de suínos e 682 amostras obtidas de humanos. Quinze suínos foram necropsiados. Amostras fecais suínas e humanas foram examinadas para detecção de ovos de *Ascaris*. Os parasitas adultos, obtidos nas necropsias, foram estudados através de microscopia eletrônica de varredura (MEV), e o gene mitocondrial codificante da enzima citocromo oxidase 1 (*cox*1) foi parcialmente amplificado e sequenciado para análises filogenéticas e de taxonomia molecular. A prevalência de *Ascaris* em amostras fecais de suínos foi 16/100 (16%), não sendo identificado nenhum caso de infecção por este parasita em humanos. A análise por MEV de parasitas recuperados de suínos demonstrou características morfológicas de *Ascaris suum*. As sequências nucleotídicas de *cox*1 foram compatíveis com *A. suum*. As sequências originais e de referência (obtidas no GeneBank) foram organizadas em *clusters* que não segregaram os parasitas por hospedeiro ou região geográfica. Os maiores haplogrupos foram dominados pelos haplótipos H01, H02 e H31. Nas comunidades estudadas, não foi evidenciada transmissão zoonótica de *A. suum* na interface suíno-humana.

## Introduction

*Ascaris* spp. (roundworms, phylum Nematoda) are helminths that inhabit the small intestine of humans and pigs (Leung et al., 2020). *Ascaris lumbricoides* infects humans and *Ascaris suum* is the parasite of swine, including the domestic pig (*Sus scrofa domesticus*) and the wild boar (*Sus scrofa scrofa*) (Alves et al., 2016). *A. lumbricoides* and *A. suum* are closely related species with a few morphological differences that are restricted to the mouthparts (lips and denticles) (Ansel & Thibaut, 1973), and genetic overlaps have led to the proposition that they represent a single species (Leles et al., 2012; Alves et al., 2016). Cross-transmission between humans and pigs is possible since *A. lumbricoides* and *A. suum* can complete the life cycle in either host (Nejsum et al., 2012; Silva et al., 2021). Whether *A. suum* evolutionarily derives from *A. lumbricoides* or *vice versa*, whether both derive from a common ancestor and evolved as distinct species in specific hosts, and the epidemiological importance of pigs as reservoirs of human infections in specific scenarios persist as knowledge gaps (Loreille & Bouchet, 2003; Leles et al., 2012; Betson et al., 2014).

Previous studies in Brazil reported infection rates with *Ascaris* in swine of 3.5% in Minas Gerais and 1.6% in São Paulo (Nishi et al., 2000). A survey in Brazil showed a high prevalence of human ascariasis in the state of Maranhão (17.5%), a state adjacent to Piauí, which has a prevalence of 3.9% in schoolchildren (Katz, 2018). Cross-sectional studies in specific communities estimated prevalences of 5.7% in Piauí, 9.3% in Maranhão, 1.4% in Rio de Janeiro, and 24.4% in Pará, demonstrating the great epidemiologic diversity in Brazil (Almeida et al., 2020; Calegar et al., 2021a, b).

In rural and peri-urban communities in the state of Piauí, most families engage in the extensive raising of pigs, whereby animals without regular deworming live in close contact with humans. The aim of the present study was to assess morphologic and genetic data on ascariasis in swine and their breeders in an environment highly contaminated by fecal matter, exploring the possibility of cross-host transmission in the state of Piauí, Brazil.

## Material and Methods

*Description of the study area.* The study was conducted in three municipalities in the state of Piauí, northeastern Brazil: Nossa Senhora de Nazaré (NSN; communities Fonte Perto, Passa Bem, Ferreiro, Jardineira, São Joaquim, Aroeira, Pereiros, Bairro de Fátima, Oiticica, and Centro), Teresina (TER; communities Acampamento 8 de Março and Assentamento 17 de Abril), and São João do Piauí (SJPI; communities Jacaré, Chiqueirinho, Lagoa da Serra). Table 1 presents the climatic, environmental and sociodemographic characteristics of the studied communities. All communities practice extensive swine farming, with the animals released into the peridomestic environment or in rudimentary pigsties located close to the houses.

**Table 1:** Climatic, environmental and sociodemographic characteristics of the surveyed communities in the municipalities of Nossa Senhora de Nazaré, São João do Piauí and Teresina, Piauí state, Brazil.

**Characteristics**	**Nossa Senhora de Nazaré** **	**São João do Piauí** ***	**Teresina** ****
***Climatic and Environmental***			
*Mean Temperature ºC (minimum-maximum)*	28.6 (23.1-37.1)	27.8 (21.9-35.6)	27.9 (22.9-36.9)
*Mean Rainfall mm (minimum-maximum)*	107.6 (2-314)	57.9 (0-149)	120.9 (3-328)
*Biome*	Cerrado	Caatinga	Cerrado
***Sociodemographic***			
*% Age group (years)*			
0 - 14	31% (127/410)	31.3% (41/131)	33.3% (47/141)
15 - 35	28.5% (117/410)	35.1% (46/131)	22% (31/141)
> 36	40.5% (166/410)	33.6% (44/131)	44.7% (63/141)
*% Open evacuation*	41% (168/410)	66.4% (87/131)	68.1% (96/141)
*% Poverty* *			
Yes	38.5% (158/410)	29.8% (39/131)	46.1% (65/141)
No	61.5% (252/410)	70.2% (92/131)	53.9% (76/141)

* Per capita monthly family income <24USD (considering the exchange rate of 1 USD = R$ 5,45);

** Communities Fonte Perto, Passa Bem, Ferreiro, Jardineira, São Joaquim, Aroeira, Pereiros, Bairro de Fátima, Oiticica, and Centro;

*** Communities Jacaré, Chiqueirinho, Lagoa da Serra;

**** Communities Acampamento 8 de Março and Assentamento 17 de Abril.

*Study design and sampling.* A total of 100 swine (*Sus scrofa domesticus*) fecal samples were obtained (86 from NSN, 6 from SJPI and 8 from TER). Parasitological surveys included 682 breeders (one fecal sample per individual); 410 in NSN, 131 in SJPI, and 141 in TER. The volunteers were recruited for the research through home visits, whereby plastic containers were delivered to collect human fecal samples and the peridomiciliary area was inspected to verify the presence of pigs and collect fecal samples in the soil after spontaneous defecation. All households in which there was pig farming in the studied communities were visited and residents were invited to participate in the study. About 50% of the invited subjects provided the fecal sample. A questionnaire was used to obtain sociodemographic data.

*Pig necropsies.* Fifteen slaughtered pigs were necropsied in NSN. All segments of each swine's digestive tract were assessed, whereby the abdominal hollow viscera were opened and the contents were examined visually and with the aid of sieving the intestinal contents. Helminth specimens were collected and a macroscopic identification of adult *Ascaris* specimens was performed. Any adult worms found were washed carefully in 0.9% saline solution and fixed in 70% alcohol for molecular and scanning electron microscopy (SEM) analysis.

*Parasitological examinations.* Porcine and human fecal samples were processed using Ritchie's technique (centrifugal sedimentation with ethyl acetate). The original fecal samples and sediment aliquots were conditioned at -20ºC until the molecular analysis.

*Scanning electron microscopy of* Ascaris spp. The denticles and buccal orifice of each specimen were microphotographed in scanning electron microscopy (SEM). The tip of the anterior region of the worms was separated, fixed in 70% alcohol, and dehydrated in increasing concentrations of ethyl alcohol and HMDS (hexamethyldisilane). Afterwards, the material was mounted on a metallic support, covered by a thin layer of gold, and observed in a Scanning Electron Microscope JEOL JSM 6390LV (JEOL Ltd., Akishima, Tokyo, Japan) using the Rudolph Barth Electron Microscopy Platform, Oswaldo Cruz Institute, Fiocruz.

*Molecular and computational analyses.* Genomic DNA was extracted using the DNeasy Blood & Tissue kit (Qiagen, Hilden, Germany), in accordance with the manufacturer's instructions. Partial cytochrome c oxidase subunit 1 (*cox*1) gene was amplified using Platinum Taq DNA polymerase (Invitrogen, Waltham, USA) with a set of three pairs of primers (Prosser et al., 2013). The PCR conditions were as follows: initial denaturation at 94 ºC for 5 min, followed by 35 cycles of 94 ºC for 40 s, 55 ºC for 40 s, 72 ºC for 1 min and a final extension at 72 ºC for 5 min. The amplicons were first purified using a DNA Illustra GFX PCR and a Gel Band Purification kit (GE HealthCare, Pittsburgh, USA) and then subjected to sequencing of both strands using the BigDye Terminator v. 3.1 cycle sequencing kit (Thermo Fisher Scientific, Foster City, USA) on an ABI 3730 automated DNA sequencer (Applied Biosystems).

The nucleotide sequences were edited using Bioedit software v.7.0.4. (Bioedit, Manchester, United Kingdom; Hall, 1999). Nucleotide similarity with GenBank sequences was verified with the Basic Local Alignment Search Tool - BLASTn (National Center for Biotechnology Information - NCBI). Orthologous reference sequences (*Ascaris* spp. n=77 and outgroups n=3) were retrieved from GenBank (Supplementary Material Table S1). Sequences with degenerate bases were not included. The sequences obtained in the present study (n=25) were deposited in GenBank under accession numbers OL960110-34.

Phylogenetic inferences were performed using the Molecular Evolutionary Genetics Analysis (MEGA) v.7.0.20 software (Kumar et al., 2016). The Maximum Likelihood (ML) method was applied using the Hasegawa-Kishino-Yano (HKY) model with non-uniformity of evolutionary rates among sites (+G). The model was selected using the Bayesian Information Criterion (BIC) in MEGA v.7.0.20 with 1,000 replicated bootstrap values (Kumar et al., 2016; Tamura & Nei, 1993; Hasegawa et al., 1985). DNA Sequence Polymorphism (DnaSP) v.6 software was used to prepare an input file (Rozas et al., 2017). A median-joining (MJ) haplotype network was constructed in Network v.5.0.0.3 software (Bandelt et al., 1999). Intraspecific genetic diversity of *Ascaris* groups were determined using Pairwise Distance in ARLEQUIN v.3.5.2.2 software (Excoffier & Lischer, 2010). The following molecular diversity indices were included: haplotype diversity (*h*); nucleotide diversity (π); haplotypes; polymorphic sites; substitutions; transitions; and transversions. DNA Sequence Polymorphism (DNASP) v.6 (Rozas et al., 2017) was used to prepare an input file.

## Results

*Ascaris* spp. *infection rates in pigs and their breeders.* The overall positivity rate for *Ascaris* eggs in the pig fecal samples was 16/100 (16%) (Table 2) (NSN 10/86 [11.6%]; SJPI 3/6 [50%]; and TER 3/8 [37.5%]). Concerning the 15 necropsied pigs, 28 *Ascaris* spp. adult specimens were recovered from three animals (one pig with 26 specimens and two with one each), and had also been positively identified in the parasitological stool. Among the 682 human fecal samples, no *Ascaris* eggs were identified in the parasitological examinations of the feces (Table 2).

**Table 2:** The overall positivity rate for *Ascaris* spp. eggs in swine and human fecal samples, Piauí, Brazil.

Municipalities	Host	
	Human	Swine
**Nossa Senhora de Nazaré**	0/410 (0%)	10/86 (11.6%)
**São João do Piauí**	0/131 (0%)	3/6 (50%)
**Teresina**	0/141 (0%)	3/8 (37.5%)
**Total**	682	100

**Figure 1:**
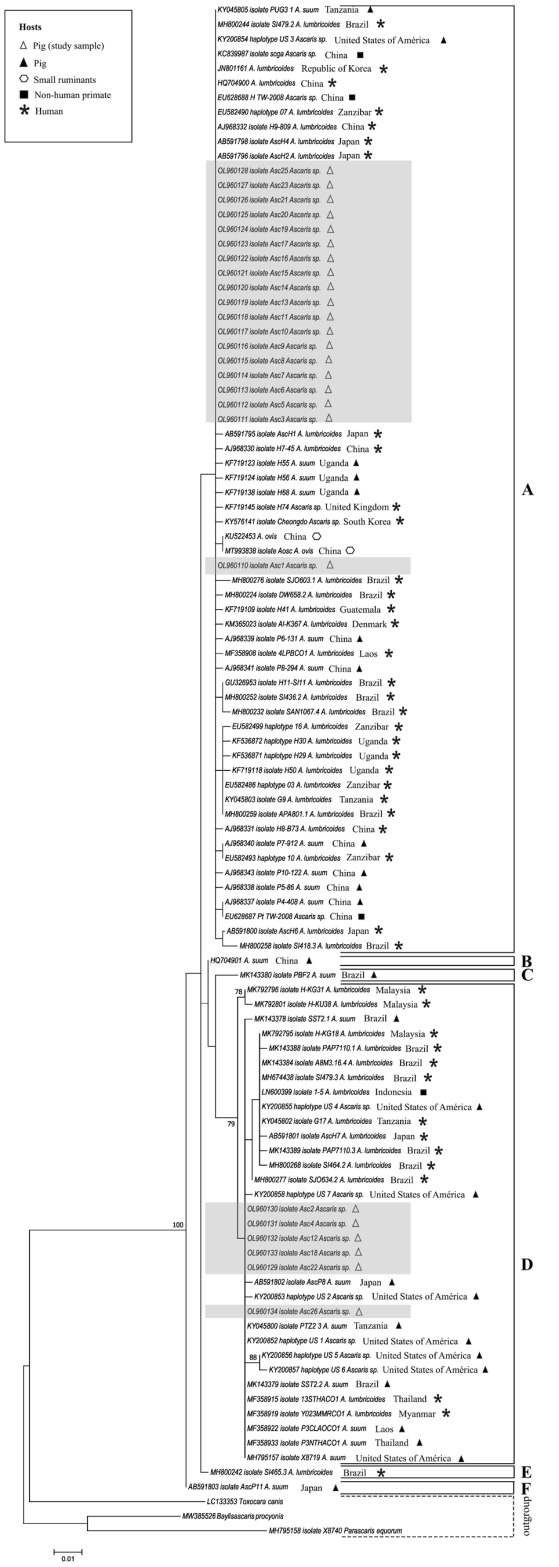
Phylogenetic tree generated from *Ascaris* spp. mitochondrial *cox*1 nucleotide sequences (383 bp). Bootstrap values >70% are reported. *Toxocara canis*, *Bayliascaris procyonis* and *Parascaris equorum* as outgroups. Further details of reference strains can be found in Supplementary Material Table S1.

**Figure 2:**
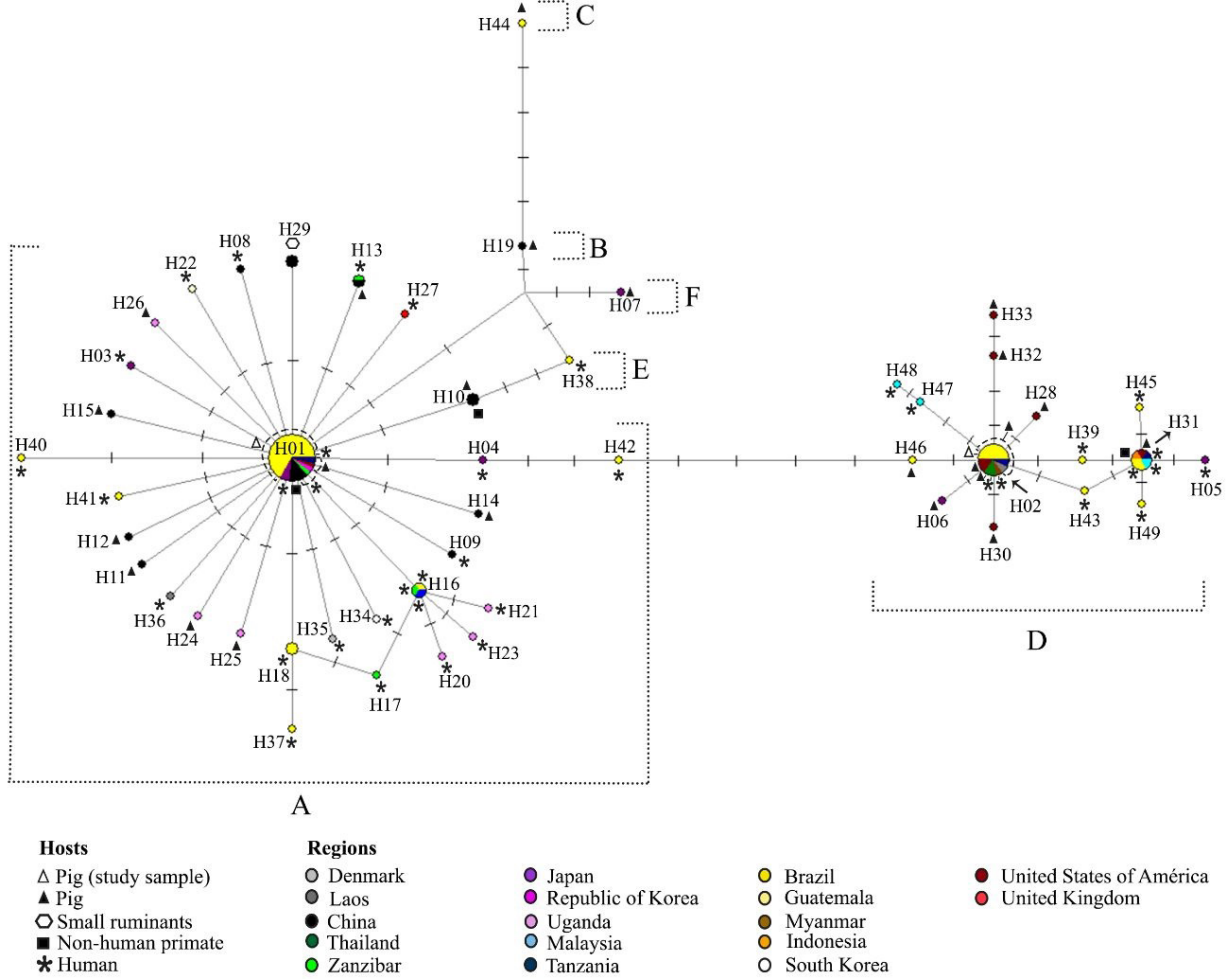
Median-joining network from *Ascaris* spp. mitochondrial *cox*1 nucleotide sequences (383 bp). The area of the circle is proportional to the sequence number.

*Genetic characterization of Ascaris* spp. All 25 *cox1* partial sequences from adult worms were obtained from a single swine host. The phylogenetic tree, which included sequences retrieved from GenBank, was organized into six main clusters (Figure 1). Cluster A grouped 19 *Ascaris* spp. original sequences that were identical to: i) *A. lumbricoides* from human hosts in Brazil, Japan, China, Republic of Korea and Zanzibar, ii) *A. suum* from swine in Uganda, iii) *Ascaris* spp. from swine in the USA, and iv) *Ascaris* spp. infecting non-human primates (gibbons) in China. Cluster B included an *A. suum* sequence from a swine in China and cluster C included an *A. suum* sequence from a swine in Brazil. Cluster D grouped six sequences from the present study with great similarity [100%] with *A. suum* from swine in Tanzania, Laos, Thailand and the USA, and *A. lumbricoides* from humans in Thailand and Myanmar. Cluster E included an *A. lumbricoides* reference sequence from humans from Brazil and cluster F included a sequence of *A. suum* from a swine from Japan. The ML tree and MJ network showed similar topologies. The sequences (orthologous and those obtained in this study) were distributed in 49 haplotypes (Figure 2). Nineteen *cox*1 sequences grouped with cluster A, representing the already described and widely distributed H01 haplotype (Figure 2). Six sequences are grouped with cluster D, which represents the H02 haplotype. Intraspecific diversity indices are shown in Table 3. We identified that the average number of nucleotide differences among genotypes (π) drawn from groups A and D is lower when compared to the entire population of *Ascaris* (all *Ascaris* group). These results suggest the presence of genetically distinct population groups within the genus *Ascaris*.

**Table 3:** Summary of diversity indexes of *Ascaris* spp. based on *cox*1 locus (383 bp).

Molecular diversity index	*Ascaris* group		
	A	D	All Ascaris*
N	65	33	102
*h* ± SD	0.787 ± 0.054	0.799 ± 0.064	0.893 ± 0.024
π ± SD	0.003 ± 0.002	0.004 ± 0.003	0.132 ± 0.007
Nº of haplotypes	30	15	49
Nº of polymorphic sites	30	14	49
Nº of substitutions	30	14	50
Nº of transitions	24	12	39
Nº of transversions	6	2	11

*h*: haplotype diversity; π: nucleotide diversity; SD: standard deviation.

* *Ascaris* groups included A, B, C, D, E, and F. Groups with 1 sequence (B, C, E, and F) were included only in “All *Ascaris*”.

**Figure 3:**
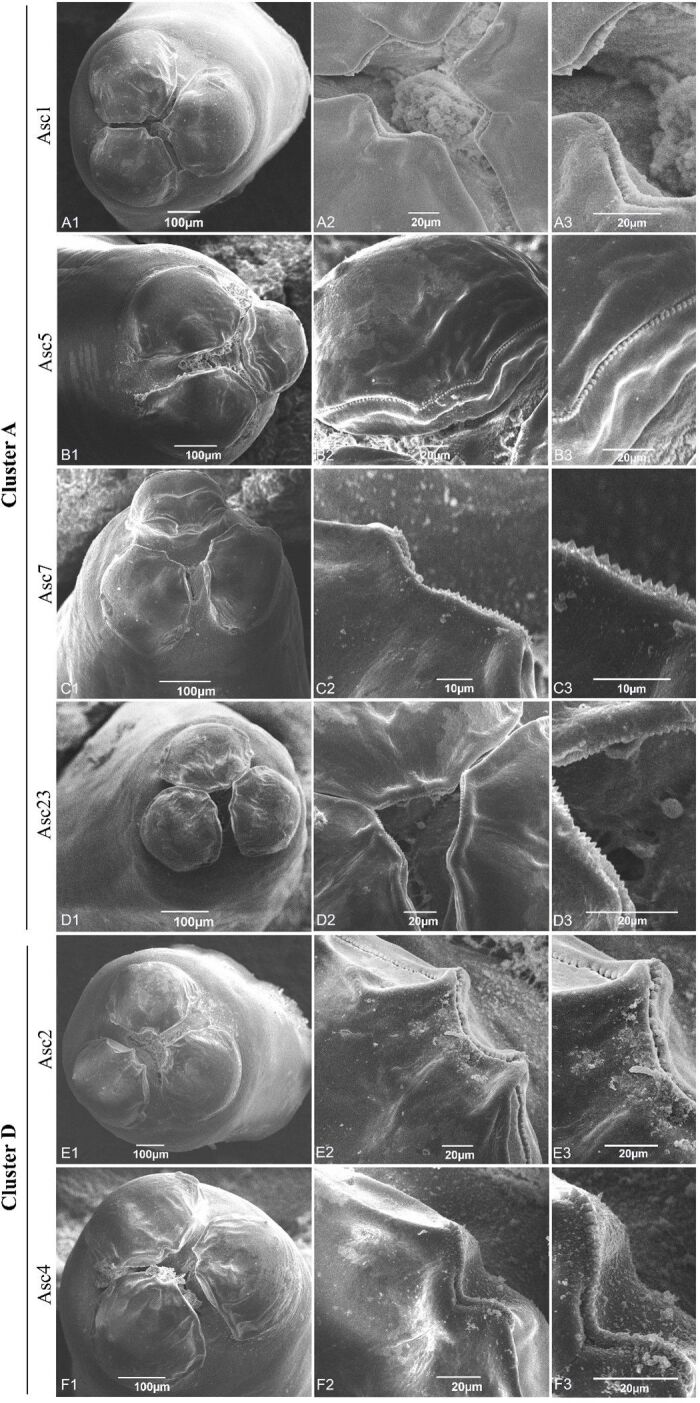
Scanning electron micrographs demonstrating the morphological characteristics of the mouthparts of adult parasites obtained by necropsy of swine. The morphology of the mouth opening and denticles is compatible with *Ascaris suum*.

*Morphological characterization of Ascaris* spp. *through scanning electron microscopy.* The analysis of i) the lips in the mouth orifice; ii) the denticles (close and pointed or blunt-shaped); iii) the slit-like groove; and iv) the sharp cavity (Figure 3) demonstrated that all six adult worms examined (four corresponding to cluster A and the H01 haplotype, and two to cluster B and the H02 haplotype) were morphologically compatible with *A. suum*.

## Discussion

The present study demonstrated *Ascaris suum* circulating among pigs in a poor sanitation background. No human infections were identified despite close contact between pigs and breeders in all communities studied. A previous study in Piauí, Brazil, revealed a low frequency of human ascariasis, probably associated with low soil moisture in the Brazilian semiarid region (Alves et al., 2003; Weaver et al., 2010). The National Survey on the Prevalence of Schistosomiasis and Soil-transmitted Helminths (2010-2015) reinforced that ascariasis is hypoendemic in human populations in the state of Piauí (Katz, 2018). Our data suggest that ascariasis is enzootic in the swine population raised extensively in this region, contradicting the argument about limited transmission due to edaphoclimatic factors. Inversely to what was observed in the present study, concomitant infection has been demonstrated in humans and pigs, in addition to the presence of eggs in the soil, suggesting zoonotic transmission cycles in some villages in China (Weidong et al., 1996). Similarly, exposure to swine on farms is a risk factor for human ascariasis in the USA and the United Kingdom (Bendall et al., 2011; Miller et al., 2015).

In the present study, the morphological analysis performed via SEM on six *Ascaris* specimens recovered from pigs showed that they were compatible with *A. suum*, in line with previously published morphological data (Madden et al., 1970; Madden & Tromba, 1976). Published data on comparative morphology of *A. suum* and *A. lumbricoides* are very scarce.

Regarding the genetic diversity explored using *cox*1, the topology of the phylogenetic tree and the MJ network followed the same pattern analyzed using previously reported mitochondrial markers, with the formation of two main groups (Monteiro et al., 2019; Easton et al., 2020; Nejsum et al., 2017; Sadaow et al., 2018). In the MJ network, the star-like shape is represented by haplogroups with a centered structure corresponding to a dominant and ancestral haplotype branched into several derived haplotypes. This profile appears to be typical of *Ascaris* spp. according to previously published data (Monteiro et al., 2019; Easton et al., 2020; Nejsum et al., 2017; Sadaow et al., 2018; Betson et al., 2012).

The largest haplogroups were dominated by haplotypes H01, H02 and H31. Interestingly, H01 and H02 were observed concomitantly in a host. These haplotypes are also widespread in the USA, Africa and Asia, circulating among swine, humans and non-human primates. In the surroundings of the dominant H01 and/or H02, the haplotypes of *Ascaris* spp., *A. ovis*, *A. lumbricoides,* and *A. suum* differed by only one polymorphism. The H01 (but not H02) haplotype has previously been described in Brazil (Monteiro et al., 2018). Other haplotypes grouped around H01 and H02 have been identified in Brazil in studies with parasites recovered from humans (Iñiguez et al., 2012; Monteiro et al., 2018; 2019.)

The phylogenetic tree and the MJ network did not reveal regional or host segregation, contrasting with population analyses using nuclear microsatellites that made this distinction, identified hybrids and suggested gene flow restriction (Criscione et al., 2007; Søe et al., 2016; Zhou et al., 2012). Although not using nuclear markers is a limitation of the present study, our results are consistent with robust research done with proteomic and genomic analysis in *A. suum* and *A. lumbricoides*, which proposes a genetic complex, or a mosaic resulting from crossover recombination (Easton et al., 2020). Our data reinforce the hypothesis that *Ascaris* spp. is a large population that is not yet differentiated and is in the process of speciation. The genetic similarity despite the variety of host species can be explained by the demographic events of the migration of peoples to the Americas and the domestication of swine since colonial times.

From a phylogenetic point of view, cross-host transmission would be supported by the high similarity of the study sequences with ascarids harbored in humans, pigs, gibbons, chimpanzees and orangutans. However, the literature on the molecular epidemiology of swine-human cross-transmission brings conflicting results, with some studies not indicating zoonotic transmission, in which swine would not act as reservoirs, and others suggesting transmission between humans and swine.

Supporting the hypothesis of no cross-host transmission, Palma et al. (2019) used PCR restriction fragment length polymorphism (PCR-RFLP) on 113 adult worms collected from children and swine in Honduras, characterizing host-specific genotypes. A review of epidemiological, molecular and experimental infection studies in China reinforced that cross-infection is limited and that pigs are not a significant source of human infection (Peng et al., 2007). Supporting zoonotic transmission, parasites recovered from human ascariasis in Asia were characterized as *A. suum* using mitochondrial and nuclear markers (Arizono et al., 2010; Zhou et al., 2012). In these two studies, parasites recovered from pigs were characterized as *A. lumbricoides*.

## Conclusion

This study demonstrates enzootic ascariasis in swine and the absence of human infections in a poor sanitation background and with close contact between animals and breeders. Specimens recovered from pigs were morphologically and genetically compatible with *A. suum*. The phylogenetic analyses of the original and GenBank *cox*1 sequences did not segregate the parasites by host or geographic region. In the communities studied, there is no evidence of the zoonotic transmission of ascariasis at the human-swine interface.

## Supplementary Material

Supplementary material accompanies this paper.

Table S1 Reference sequences of mitochondrial *cox*1 used in this study (n=80)

This material is available as part of the online article from https://doi.org/10.1590/S1984-29612023057
