# Supplementary material for: A molecular and morphological study of Ascaris suum in a human-pig contact scenario in northeastern Brazil
Source: Rev Bras Parasitol Vet. 2023 Oct 13;32(3):e005623. doi: 10.1590/S1984-29612023057 (PMC10706455; doi:10.1590/S1984-29612023057)
Supplement: Table S1 [file rbpv-32-3-e005623-Suppl.pdf]

Supplementary Table S1

Reference sequences of mitochondrial *cox*1 used in this study (n=80).

Continent	GenBank accession number	Species	Isolate/ haplotype	Host	Country	Haplotype	Reference
Americas	MH800224	*A. lumbricoides*	DW658.2	Human	Brazil	H41	[2]
	MH800232	*A. lumbricoides*	SAN1067.4	Human	Brazil	H37	[2]
	MH800242	*A. lumbricoides*	SI465.3	Human	Brazil	H38	[2]
	MH800244	*A. lumbricoides*	SI479.2	Human	Brazil	H01	[2]
	MH800252	*A. lumbricoides*	SI436.2	Human	Brazil	H18	[2]
	MH800258	*A. lumbricoides*	SI418.3	Human	Brazil	H42	[2]
	MH800259	*A. lumbricoides*	APA801.1	Human	Brazil	H16	[2]
	MH800268	*A. lumbricoides*	SI464.2	Human	Brazil	H43	[2]
	MH800276	*A. lumbricoides*	SJO603.1	Human	Brazil	H40	[2]
	MH800277	*A. lumbricoides*	SJO634.2	Human	Brazil	H39	[2]
	MH674438	*A. lumbricoides*	SI479.3	Human	Brazil	H31	[2]
	MK143378	*A. suum*	SST2.1	Pig	Brazil	H46	[18]
	MK143379	*A. suum*	SST2.2	Pig	Brazil	H01	[18]
	MK143380	*A. suum*	PBF2	Pig	Brazil	H44	[18]
	MK143384	*A. lumbricoides*	A8M3.16.4	Human	Brazil	H31	[18]
	MK143388	*A. lumbricoides*	PAP7110.1	Human	Brazil	H49	[18]
	MK143389	*A. lumbricoides*	PAP7110.3	Human	Brazil	H45	[18]
	GU326953	*A. lumbricoides*	H11-SI11	Human	Brazil	H18	[16]
	KF719109	*A. lumbricoides*	H41	Human	Guatemala	H22	[11]
	KY045805	*A. suum*	PUG3	Pig	Tanzania	H01	[1]
	KY200852	*Ascaris* sp.	US 1	Pig	USA	H02	[3]
	KY200853	*Ascaris* sp.	US 2	Pig	USA	H28	[3]
	KY200854	*Ascaris* sp.	US3	Pig	USA	H01	[3]
	KY200855	*Ascaris* sp.	US 4	Pig	USA	H31	[3]

	KY200856	*Ascaris* sp.	US 5	Pig	USA	H32	[3]
	KY200857	*Ascaris* sp.	US 6	Pig	USA	H33	[3]
	KY200858	*Ascaris* sp.	US 7	Pig	USA	H30	[3]
	MH795157	*A. suum*	X8719	Pig	USA	H02	[19]
	MH795158	*Parascaris equorum***	X8740	Horse	USA	-	[19]
	MW385526	*Baylisascaris procyonis***	-	Raccoon	USA	-	[21]
Asia	KC839987	*Ascaris* sp.	SCGA	Gibbon	China	H01	[4]
	HQ704900	*A. lumbricoides*	-	Human	China	H01	[6]
	HQ704901	*A. suum*	-	Pig	China	H19	[6]
	EU628688	*Ascaris* sp.	H TW-2008	Gibbon	China	H01	[7]
	AJ968330	*A. lumbricoides*	H7-45	Human	China	H08	[9]
	AJ968331	*A. lumbricoides*	H8-B73	Human	China	H09	[9]
	AJ968332	*A. lumbricoides*	H9-809	Human	China	H01	[9]
	AJ968337	*A. suum*	P4-408	Pig	China	H10	[9]
	AJ968338	*A. suum*	P5-86	Pig	China	H11	[9]
	AJ968339	*A. suum*	P6-131	Pig	China	H12	[9]
	AJ968340	*A. suum*	P7-912	Pig	China	H13	[9]
	AJ968341	*A. suum*	P8-294	Pig	China	H14	[9]
	AJ968343	*A. suum*	P10-122	Pig	China	H15	[9]
	KU522453	*A. ovis*	-	Small ruminants	China	H29	*
	MT993838	*A. ovis*	Aosc	Sheep	China	H29	[13]
	EU628687	*Ascaris* sp.	Pt1 TW-2008	Chimpanzee	China	H10	[4]
	JN801161	*A. lumbricoides*	-	Human	Republic of Korea	H01	[5]
	KY576141	*Ascaris* sp.	Cheongdo	Human	South Korea	H34	[12]
	AB591795	*A. lumbricoides*	AscH1	Human	Japan	H03	[10]
	AB591796	*A. lumbricoides*	AscH2	Human	Japan	H01	[10]
	AB591798	*A. lumbricoides*	AscH4	Human	Japan	H01	[10]
	AB591800	*A. lumbricoides*	AscH6	Human	Japan	H04	[10]
	AB591801	*A. lumbricoides*	AscH7	Human	Japan	H05	[10]
	AB591802	*A. suum*	AscP8	Pig	Japan	H06	[10]
	AB591803	*A. suum*	AscP11	Pig	Japan	H07	[10]

	MF358908	*A. lumbricoides*	4LPBCO1	Human	Laos	H36	[15]
	MF358922	*A. suum*	P3CLAOCO1	Pig	Laos	H02	[15]
	MK792796	*A. lumbricoides*	H-KG31	Human	Malaysia	H47	*
	MK792801	*A. lumbricoides*	H-KU38	Human	Malaysia	H48	*
	MK792795	*A. lumbricoides*	H-KG18	Human	Malaysia	H31	*
	MF358919	*A. lumbricoides*	Y023MMRCO1	Human	Myanmar	H02	[15]
	LN600399	*A. lumbricoides*	1-5	Orangutan	Indonesia	H31	*
	MF358915	*A. lumbricoides*	13STHACO1	Human	Thailand	H02	[15]
	MF358933	*A. suum*	P3NTHACO1	Pig	Thailand	H02	[15]
	LC133353	*Toxocara canis***	-	Dog	Viet Nam	-	[20]
Africa	EU582490	*A. lumbricoides*	07	Human	Zanzibar	H01	[8]
	KF719123	*A. suum*	H55	Pig	Uganda	H24	[11]
	KF719124	*A. suum*	H56	Pig	Uganda	H25	[11]
	KF719138	*A. suum*	H68	Pig	Uganda	H26	[11]
	EU582499	*A. lumbricoides*	16	Human	Zanzibar	H17	[8]
	KF536872	*A. lumbricoides*	H30	Human	Uganda	H21	[17]
	KF536871	*A. lumbricoides*	H29	Human	Uganda	H20	[17]
	KF719118	*A. lumbricoides*	H50	Human	Uganda	H23	[11]
	EU582486	*A. lumbricoides*	03	Human	Zanzibar	H16	[8]
	KY045803	*A. lumbricoides*	G9	Human	Tanzania	H16	[1]
	EU582493	*A. lumbricoides*	10	Human	Zanzibar	H13	[8]
	KY045802	*A. lumbricoides*	G17	Human	Tanzania	H31	[1]
	KY045800	*A. suum*	PTZ2_3	Pig	Tanzania	H02	[1]
Europe	KM365023	*A. lumbricoides*	Al-K367	Human	Denmark	H35	[14]
	KF719145	*Ascaris* sp.	H74	Human	United Kingdom	H27	[11]

*Unpublished. ** Outgroup.

References

1 Nejsum P, Hawash MB, Betson M, et al. *Ascaris* phylogeny based on multiple whole mtDNA genomes. Infect Genet Evol. 2017;48:4-9.

2 Monteiro KJL, Calegar DA, Amazonas Research Group, et al. Kato-Katz thick smears as a DNA source of soil-transmitted helminths. J Helminthol. 2018;94:e10.

3 Jesudoss Chelladurai J, Murphy K, Snobl T, et al. Molecular epidemiology of *Ascaris* infecting pigs in Iowa, USA. J Infect Dis. 2017;215(1):131-8

4 Xie Y, Niu L, Zhao B, et al. Complete mitochondrial genomes of chimpanzee-and gibbon-derived *Ascaris* isolated from a zoological garden in southwest China. PLoS One. 2013;8(12):e82795.

5 Park YC, Kim W, Park JK. The complete mitochondrial genome of human parasitic roundworm, *Ascaris lumbricoides*. Mitochondrial DNA. 2011;22(4):91-3.

6 Liu GH, Wu CY, Song HQ, et al. Comparative analyses of the complete mitochondrial genomes of *Ascaris lumbricoides* and *Ascaris suum* from humans and pigs. Gene. 2012;492(1):110-16.

7 Niu LL, Chen SJ, Wang T, et al. Genetic relationship of ascarid nematodes from giant panda and seven other species of captive wild mammals based on COXI and COXII genes. Acta Vet et Zootec Sinica. 2012;43:1645-50.

8 Betson M, Halstead FD, Nejsum P, et al. A molecular epidemiological investigation of *Ascaris* on Unguja, Zanzibar using isoenyzme analysis, DNA barcoding and microsatellite DNA profiling. Trans R Soc Trop Med Hyg. 2011;105(7):370-9.

9 Peng W, Yuan K, Hu M, et al. Mutation scanning-coupled analysis of haplotypic variability in mitochondrial DNA regions reveal slow gene flow between human and porcine *Ascaris* in endemic regions of China. Electrophoresis. 2005;26(22):4317-26.

10 Arizono N, Yoshimura Y, Tohzaka N, et al. Ascariasis in Japan: is pig-derived *Ascaris* infecting humans? Jpn J Infect Dis. 2010;63(6):447-8.

11 Betson B, Nejsum P, Bendall RP, et al. Molecular epidemiology of ascariasis: a global perspective on the transmission dynamics of Ascaris in people and pigs. J Infect Dis. 2014;210(6):932-41.

12 Hong JH, Oh CS, Seo M, et al. Ancient *Ascaris* DNA sequences of cytochrome B, cytochrome C oxidase subunit 1, NADH dehydrogenase subunit 1, and internal transcribed spacer 1 genes from Korean Joseon mummy feces. J Parasit. 2017;103(6):795-800.

13 Chen Y, Wang L, Zhou X, et al. The mitochondrial genome of the sheep roundworm *Ascaris ovis* (Ascaridida: Nematoda) from Southwest China. Mitochondrial DNA B Resour. 2021;6(2):410-412.

14 Søe MJ, Nejsum P, Fredensborg BL, et al. DNA typing of ancient parasite eggs from environmental samples identifies human and animal worm infections in Viking-age settlement. J Parasit.2015;101(1):57-63.

15 Sadaow L, Sanpool O, Phosuk I, et al. Molecular identification of *Ascaris lumbricoides* and *Ascaris suum* recovered from humans and pigs in Thailand, Lao PDR, and Myanmar. Parasitol Res. 2018;117(8):2427-36.

16 Iñiguez AM, Leles D, Jaeger LH, et al. Genetic characterisation and molecular epidemiology of *Ascaris* spp. from humans and pigs in Brazil. Trans R Soc Trop Med Hyg. 2012;106(10):604-12.

17 Betson M, Nejsum P, Llewellyn-Hughes J, et al. Genetic diversity of Ascaris in southwestern Uganda. Trans R SocTrop Med Hyg. 2012;106(2):75-83.

18 Monteiro KJL, Calegar DA, Santos JP, et al. Genetic diversity of Ascaris spp. infecting humans and pigs in distinct Brazilian regions, as revealed by mitochondrial DNA. PLoS ONE. 2019;14(6):e0218867.

19 Camp LE, Radke MR, Shihabi DM, et al. Molecular phylogenetics and species-level systematic of *Baylisascaris*. Int J Parasitol Parasites Wildl. 2018;7(3):450-62.

20 Quyen NT, Lan NTK, Doanh PN. Molecular phylogenetic relationship of *Toxocara canis* isolated from dogs in Phu Tho province, Vietnam. Acad J Biol. 2016;38(2):140-145.

21 Carlson CR, Schutz CL, Pagan C, et al. Phylogeography of *Baylisascaris procyonis* (raccoon roundworm) in North America. J Parasitol. 2021;107(3):411-20.
